# **Cognitive and anatomical data in a healthy cohort of adults**

**DOI:** 10.1016/j.dib.2016.03.100

**Published:** 2016-04-05

**Authors:** P.D. Watson, E.J. Paul, G.E. Cooke, N. Ward, J.M. Monti, K.M. Horecka, C.M. Allen, C.H. Hillman, N.J. Cohen, A.F. Kramer, A.K. Barbey

**Affiliations:** a Beckman Institute for Advanced Science and Technology, University of Illinois at Urbana–Champaign, Urbana, IL, USA; b Department of Kinesiology and Community Health, University of Illinois at Urbana–Champaign, Urbana, IL, USA; c Department of Psychology, University of Illinois at Urbana–Champaign, Champaign, IL, USA; d Decision Neuroscience Laboratory, University of Illinois at Urbana–Champaign, Champaign, IL, USA; e Department of Bioengineering, University of Illinois at Urbana–Champaign, Urbana, IL, USA; f Department of Internal Medicine, University of Illinois at Urbana–Champaign, Champaign, IL, USA; g Department of Speech and Hearing Science, University of Illinois at Urbana–Champaign, Champaign, IL, USA; h Neuroscience Program, University of Illinois at Urbana–Champaign, Champaign, IL, USA

**Keywords:** Independent component analysis, Fluid intelligence, Neuroanatomy, Tractography, Individual differences

## Abstract

We present data from a sample of 190 healthy adults including assessments of 4 cognitive factor scores, 12 cognitive tests, and 115 MRI-assessed neuroanatomical variables (cortical thicknesses, cortical and sub-cortical volumes, fractional anisotropy, and radial diffusivity). These data were used in estimating underlying sources of individual variation via independent component analysis (Watson et al., In press) [25].

**Specifications Table**

**Table:** 

Subject area	*Neuroscience*
More specific subject area	*Anatomical Neuroimaging*
Type of data	*Table of cognitive testing data and MRI assessed structural data.*
How data was acquired	*Cognitive testing, Freesurfer automated segmentation of T1 weighted 3D MPRAGE images on a Siemens Magnetom Trio 3T whole-body MRI*
Data format	*Analyzed*
Experimental factors	*Brief description of any pretreatment of samples*
Experimental features	*Multi-modal MRI collection prior to a large cognitive training intervention.*
Data source location	*Urbana, Illinois*
Data accessibility	*Public repository: Open Science framework INSIGHT project: https://osf.io/9ezwc/*

**Value of the data**

• These data characterize individual variation across demographic, neuroanatomical, and cognitive factors.
• These provide a useful model of individual variation that can be used to control for individual differences.
• The relationship between these data and other neuroimaging (such as resting state) and cognitive data remains unexplored and would be a fruitful area of collaboration.
• These data can be used to estimate patterns of joint variance across and within different neuroimaging and behavioral methods.
• These patterns can be used to test specific cognitive–anatomical linkages.

## 1 Data

The data (Supplementary Table 1) includes cognitive and anatomical variables collected prior to a large, multi-modal cognitive training study [25]. They include:

a) Demographic measures (i.e., age, sex, and education).
b) Cardiovascular fitness measures.
c) 4 cognitive factors estimated via structural equation modeling [15].
d) Scores from the battery of 12 cognitive tests used to estimate these factors.
e) 35 cortical thickness estimates and volume estimates for these same regions.
f) 11 sub-cortical volumetric estimates.
g) Total brain and total intracranial volume estimates.
h) 7 estimates of ventricular size.
i) 5 estimates of corpus callosum.
j) 12 estimates of fractional anisotropy and in matter tracts.
k) 12 estimates of radial diffusivity in white matter tracts.

## 2 Experimental design, materials and methods

### 2.1 Demographics

The 190 participants consisted of 85 females, and 105 males. The age range in our sample was 18–44 years, with a median of 22 years, and a mean of 24.3 years. The mean educational level of the participants was “some college” (i.e., median score 3, mean score 3.6) as reported on a scale from 1 to 5, where 1 denoted “less than a high school diploma”, 2 denoted “high school diploma or equivalent”, 3 denoted “some college”, 4 denoted “college degree”, and 5 denoted “post-graduate education.”

### 2.2 Aerobic fitness assessment

Maximal oxygen consumption (VO_2max_) was measured using a computerized indirect calorimetry system (ParvoMedics True Max 2400) and a modified Balke protocol [1] with averages for oxygen uptake (VO2) and respiratory exchange ratio (RER) assessed every 20 s. Participants ran on a motor-driven treadmill at a constant speed, with 2.0% increases in grade every two minutes until volitional exhaustion. The raw value was adjusted for body size, age, and gender to produce a VO_2max_ percentile score.

### 2.3 Cognitive tests and factor scores

Participants received a battery of 12 cognitive tests designed to estimate underlying latent variables corresponding to cognitive constructs (see Table 1). The four latent variables of interest were fluid intelligence (gf), working memory (wm), executive function (ef), and episodic memory (em). Each of these latent variables was measured with three cognitive tests as follows. Fluid intelligence (gf) was measured by the BOMAT, number series, and letter sets tests [3,4,7]. Working memory (wm) was measured by the reading, rotation, and symmetry span tests [8,23]. Executive function (ef) was measured by the Garavan, Keep Track, and Stroop tests [14], [22], [26]. Episodic memory (em) was measured by immediate free recall, words, pictures and paired associates tests [23], [24], [9]. Using a structural equation modeling approach [15], across the larger sample of 518 participants, we extracted estimates of the four cognitive construct latent variables (i.e., gf, wm, ef, em). Because Garavan and Stroop produce error scores, while all others are measures of accuracy, we inverted these two values (i.e., multiplied by −1) in order to ensure all cognitive variables had the same sign.

**Table 1:** Included measures.

Data categories	Specific measures
Demographics & cardiovascular fitness	Age
	Years of education
	Sex
	VO_2max_ percentile
Cognition	Fluid intelligence (fluid g)
	Working memory (wm)
	Executive function (ef)
	Episodic memory (em)
	BOMAT (correct trials)
	Number series (correct trials)
	Letter Sets (correct trials)
	Reading span
	Rotation span
	Symmetry span
	Garavan (inverse total errors)
	Keep Track Words Recalled
	Stroop (inverse cost)
	Immediate free recall Words
	Immediate free recall Pictures
	Immediate free recall Paired
	Associates
Cortical thicknesses	Superior parietal
	Postcentral
	Precuneus
	Lateral occipital
	Mean cortical thickness
	Superior temporal
	Inferior parietal
	Paracentral
	Precentral
	Middle temporal
	Banks of superior temporal sulcus
	Insula
	Superior frontal
	Supramarginal
	Transverse temporal
	Rostral middle frontal
	Caudal middle frontal
	Pars triangularis
	Pars opercularis
	Lateral orbitofrontal
	Pars orbitalis
	Frontal pole
	Posterior cingulate
	Inferior temporal
	Cuneus
	Peri calcarine
	Rostral anterior cingulate
	Medial orbitofrontal
	Caudal anterior cingulate
	Isthmus cingulate
	Fusiform
	Temporal pole
	Lingual
	Entorhinal
	Parahippocampal
Cortical volumes	Middle temporal
	Inferior parietal
	Inferior temporal
	Rostral anterior cingulate
	Posterior cingulate
	Rostral middle frontal
	Superior frontal
	Precentral
	Supra marginal
	Lateral orbitofrontal
	Fusiform
	Precuneus
	Insula
	Medial orbitofrontal
	Postcentral
	Superior temporal
	Caudal middle frontal
	Paracentral
	Superior parietal
	Isthmus cingulate
	Lateral occipital
	Transverse temporal
	Pars orbitalis
	Pars opercularis
	Caudal anterior cingulate
	Pars triangularis
	Entorhinal
	Temporal pole
	Parahippocampal
	Frontal pole
	Peri calcarine
	Cuneus
	Lingual
Sub-cortical volumes	Total Brain volume
	Total Intracranial Volume
	Hippocampus
	Ventral Diencephalon
	Cerebellum Cortex
	Cerebellum White Matter
	Thalamus
	Brain Stem
	Amygdala
	Putamen
	Accumbens area
	Pallidum
	Caudate
Ventricles	Surface Holes
	Lateral Ventricle
	Choroid plexus
	Third Ventricle
	Cerebrospinal fluid
	Inferior Lateral Ventricle
	Fourth Ventricle
Corpus callosum	CC Posterior
	CC Mid Posterior
	CC Central
	CC Mid Anterior
	CC Anterior
White matter tractography (Fractional Anisotropy)	Inferior fronto-occipital fasciculus
	Superior longitudinal fasciculus
	Temporal superior longitudinal fasciculus
	Inferior longitudinal fasciculus
	Anterior thalamic radiation
	Forceps minor
	Uncinate fasciculus
	Cingulum bundle
	Corticospinal tract
	Forceps major
	Hippocampal cingulum bundle
White matter tractography (Radial Diffusivity)	Inferior fronto-occipital fasciculus
	Superior longitudinal fasciculus
	Temporal superior longitudinal fasciculus
	Inferior longitudinal fasciculus
	Anterior thalamic radiation
	Forceps minor
	Uncinate fasciculus
	Cingulum bundle
	Corticospinal tract
	Forceps major
	Hippocampal cingulum bundle

### 2.4 Structural MRI protocol

High resolution T1-weighted brain images were acquired using a 3D MPRAGE (Magnetization Prepared Rapid Gradient Echo Imaging) protocol with 192 contiguous axial slices, collected in ascending fashion parallel to the anterior and posterior commissures, echo time (TE)=2.32 ms, repetition time (TR)=1900 ms, field of view (FOV)=230 mm, acquisition matrix 256 mm×256 mm, slice thickness=0.90 mm, and flip angle=9°. All images were collected on a Siemens Magnetom Trio 3T whole-body MRI scanner.

### 2.5 Automated volumetrics, cortical thickness estimates, and white-matter tractography

Automated brain tissue segmentation and reconstruction of the T1-weighted structural MRI images were performed using the standard recon-all processing pipeline in FreeSurfer, version 5.2.0 (Released May, 2013; http://surfer-nmr.mgh.harvard.edu/). This produced estimates of 1) cortical thickness, 2) cortical volumes, 3) sub-cortical volumes, 4) ventricles, and 5) corpus callosum [5], [6], [10], [11], [12], [13]. Segmentations and tractography were manually checked for errors. Estimates in the left and right hemispheres were summed to produce bilateral estimates, and all values were converted to *z*-scores to control for differences in scale. A complete list of estimated structures appears in Table 1. FreeSurfer produced automated segmentation that closely approximates hand tracing, but like all segmentation procedures may introduce systematic bias.

The diffusion tensor imaging estimates for fractional anisotropy (FA) and radial diffusivity (RD) data was analyzed using tract-based spatial statistics in FSL [19], [20], [21]. This pipeline involves fitting a tensor model to the raw diffusion data using fMRIDB׳s diffusion toolbox, and non-brain tissues were removed using FSL׳s brain extraction tool. All subjects׳ FA data were then aligned into a common space using the nonlinear registration tool FNIRT [18], [2]. Next, the mean FA image was created and thinned to create a mean FA skeleton that represents the centers of all tracts common to the group. Each subject׳s aligned FA data was then projected onto this skeleton to create an estimate of the subject-level value associated with each tract.
