# Supplementary material for: Cognitive and anatomical data in a healthy cohort of adults
Source: Data Brief. 2016 Apr 5;7:1221–7. doi: 10.1016/j.dib.2016.03.100 (PMC5540669; doi:10.1016/j.dib.2016.03.100)
Supplement: Supplementary file 2 — Supplementary material [file mmc2.doc]

**Conflicts of interest**

**Cognitive and anatomical data in a healthy cohort of adults.**

Watson, P.D.^1^, Paul, E. J.^1^, Cooke, G. E.^1^, Ward, N.^1^, Monti, J.M.^1^, Horecka, K.M.^1^, Allen, C.^1^, Hillman, C.H.^1,2^, Cohen, N.J.^1^, Kramer, A.F.^1^, & Barbey A.K.^1^

**Conflicts of interest: None**
